# Testing and vaccination to reduce the impact of COVID-19 in nursing homes: an agent-based approach

**DOI:** 10.1186/s12879-022-07385-4

**Published:** 2022-05-19

**Authors:** José P. Gómez Vázquez, Yury E. García, Alec J. Schmidt, Beatriz Martínez-López, Miriam Nuño

**Affiliations:** 1 Center for Animal Disease Modeling and Surveillance, University of California Davis, Davis, CA USA; 2 Department of Public Health Sciences, University of California Davis, Davis, CA USA

**Keywords:** Nursing homes, Testing, Vaccine, COVID-19, Agent-based model

## Abstract

**Background:**

Efforts to protect residents in nursing homes involve non-pharmaceutical interventions, testing, and vaccine. We sought to quantify the effect of testing and vaccine strategies on the attack rate, length of the epidemic, and hospitalization.

**Methods:**

We developed an agent-based model to simulate the dynamics of SARS-CoV-2 transmission among resident and staff agents in a nursing home. Interactions between 172 residents and 170 staff based on data from a nursing home in Los Angeles, CA. Scenarios were simulated assuming different levels of non-pharmaceutical interventions, testing frequencies, and vaccine efficacy to reduce transmission.

**Results:**

Under the hypothetical scenario of widespread SARS-CoV-2 in the community, 3-day testing frequency minimized the attack rate and the time to eradicate an outbreak. Prioritization of vaccine among staff or staff and residents minimized the cumulative number of infections and hospitalization, particularly in the scenario of high probability of an introduction. Reducing the probability of a viral introduction eased the demand on testing and vaccination rate to decrease infections and hospitalizations.

**Conclusions:**

Improving frequency of testing from 7-days to 3-days minimized the number of infections and hospitalizations, despite widespread community transmission. Vaccine prioritization of staff provides the best protection strategy when the risk of viral introduction is high.

**Supplementary Information:**

The online version contains supplementary material available at 10.1186/s12879-022-07385-4.

## Introduction

COVID-19 has highlighted many inadequacies in the American healthcare system. Elderly and frail residents of long-term care facilities (LTCFs) have experienced a disproportionate burden of infection and death. Approximately 5% of all US cases have occurred in LTCFs, yet deaths related to COVID-19 in these facilities account for 34% of all US deaths as of February 12, 2021, according to the New York Times [1]. Nationwide, there are about 44,736 LTCFs in the United States, 15,116 of which are nursing homes. Together these facilities encompass more than 1.2 million staff and 2.1 million residents based on 2015–2016 estimates [2].

Many oversight groups offered guidance on the prevention and mitigation of COVID-19 in LTCFs, including the Centers for Disease Control and Prevention (CDC) and the Center for Medicare and Medicaid Services (CMS). Substantial numbers of transmission events from symptom-free individuals made it clear that universal testing, regardless of symptoms, was a critical component of a robust prevention program [3–5]. Testing frequency was widely debated, as LTCFs had to balance the obvious need with the high cost and low availability of testing, especially early in the pandemic [5–7]. Vaccines are effective at preventing infection, serious illness and death. Despite the numerous challenges involved in the administration of vaccines, it is clear that their role has been instrumental in reducing the risk of outbreaks in LTCFs.

Nursing home residents are a priority group for vaccination, as are health care workers. The CDC launched the Pharmacy Partnerships for Long-Term Care Program in an effort to provide on-site vaccination to residents and staff members in LTCFs [8]. Though deployment of vaccines in LTCFs appears successful thus far, there is a growing concern that insufficient levels of vaccine coverage will be reached. As of the end of January 2021, median first dose rates among LTCF residents is 77.8%, but only a median of 37.5% of staff have received at least their first dose [9]. It is unclear at this time whether the lower vaccination rates among staff is a result of prioritization of residents, lack of recording alternative sources of vaccination, or staff choice; however, a survey of nursing home staff conducted in the state of Indiana (November 2020) found that 45% of respondents were willing to receive a COVID-19 vaccine immediately once available, and an additional 24% would consider it in the future [10]. While visitors are disallowed and residents only interact directly with a small number of other people, staff are the primary vector for viral introduction [11, 12]; therefore, low rates of vaccine uptake among staff should be of great concern from the perspective of preventing an outbreak. Additionally, there is limited evidence about the ability of vaccines to reduce asymptomatic transmission. Preliminary data from the UK suggests a 49.3% reduction in infections from an asymptomatic source [13]. Recent evidence of the circulation of more transmissible SARS-CoV-2 variants also raises concerns about the course of this pandemic, particularly as less than 22% of the US population have received the full vaccine dosage [14].

Previous simulation studies have been developed to study transmission dynamics of COVID-19 in LTCFs and the effect of interventions such as: different testing strategies [15–19], vaccine efficacy and distribution [20, 21], and use of PPE and other non-pharmaceutical interventions (NPIs) [21, 22]. There is a strong consensus that combined interventions with a strong focus on early detection is required to mitigate transmission.

Given the continued challenge of implementing robust protective measures in LTCFs, the bevy of unknowns around vaccine deployment, the uncertainty involved with new circulating strains, and the impending lifting of co-recreation and visitor restrictions as states ease recommendations. We developed a agent-based model (ABM) with the objective to quantify the effect of testing frequency and differing vaccination strategies on morbidity and mortality in a long term care setting, using a nursing home in Los Angeles, CA as the foundation for our model. ABMs allow for incorporating population characteristics and implementing interventions at the individual level. This is particularly useful to model COVID-19 spread, given that is well known the role of super spreaders and heterogeneity of the population in disease spread events [23], something that can be very challenging to capture using other population-based models. Our study assumes the continued presence of NPIs such as mask mandates for staff and universal testing and varies the risk of introduction by staff. The main outcome of this study is a model that can be adapted/modified to study the effects of these interventions in varied nursing home settings. Such modeling approaches can provide valuable insight into the design and deployment of combined vaccine and surveillance interventions before primary prospective research can be implemented [12].

To our knowledge, this is the first COVID-19 agent-based model developed specifically to study LTCFs in California and the role of vaccination, testing, PPE use, and their interactions for disease control.

## Methods

### Model structure

**Fig. 1:**
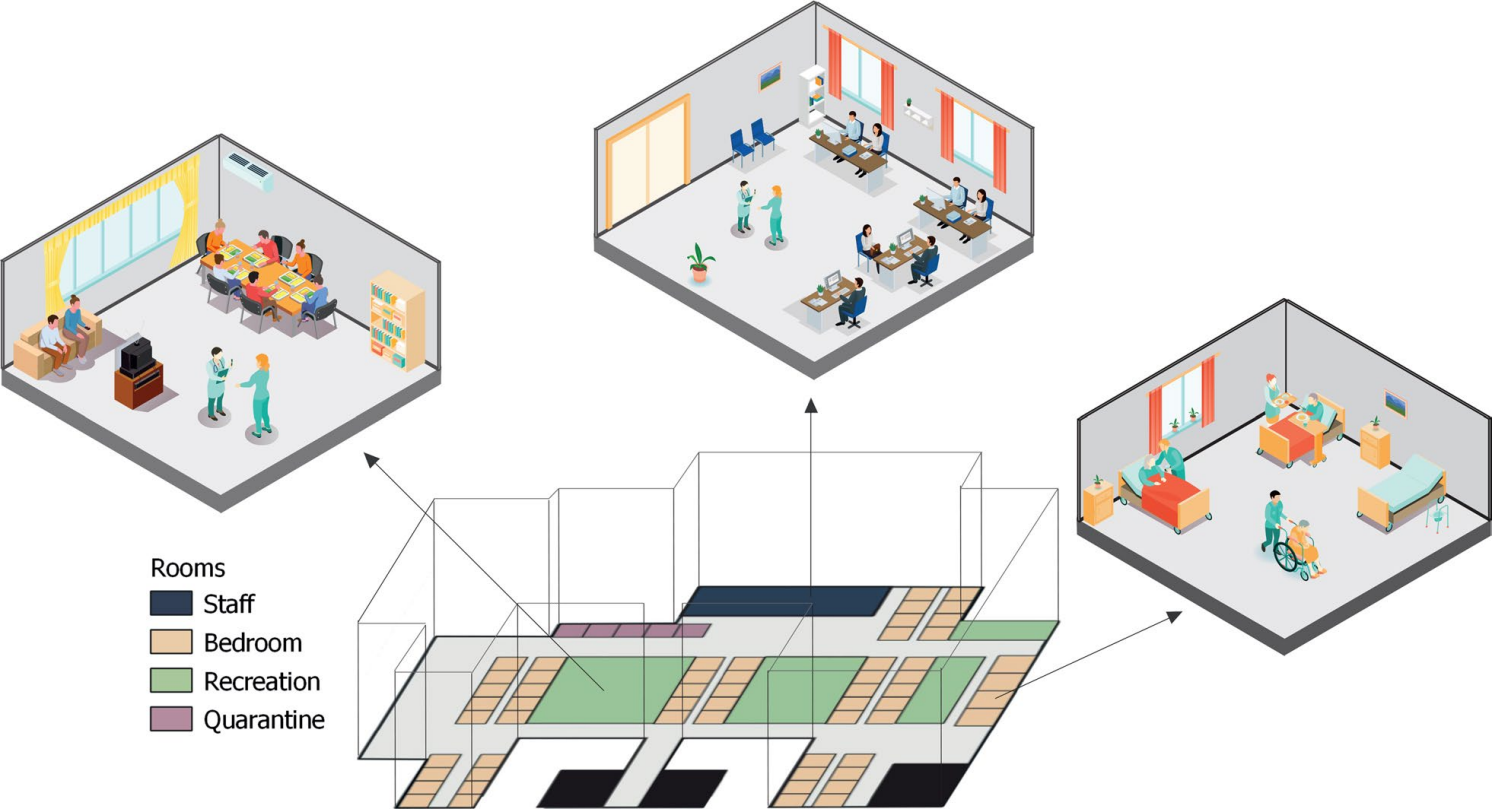
Case study of a nursing home in Los Angeles, CA

We developed a stochastic agent-based model to simulate the spread of SARS-CoV-2 in an LTCF, based on the floor plan and occupancy of a nursing home in Los Angeles County, California with 172 residents and 170 staff (Fig. 1). Our model simulates the day-to-day dynamics in a facility with a hourly time step. The simplified floor map shows the location of bedrooms with a capacity of 3 residents, 5 quarantine rooms reserved for residents with frequent outside traffic and/or capacity to quarantine detected residents, recreation areas which are currently off limits to resident and staff interactions, and rooms for staff. A detailed description of the model was described according to the ODD protocol [24] and presented in the additional section.

Agents in the model include residents and staff, with the natural history of COVID-19 captured through seven epidemiological classes (Additional file 1: Fig. S1). The model assumes that residents are not replaced with new susceptible agents, and staff with confirmed exposure to the virus are replaced by new staff confirmed negative for SARS-CoV-2 during the period of simulation. Recovered people gain immunity to reinfection lasting 120 days, and the latency period is sampled from a logarithmic normal distribution [25]. Parameters and sources are described in Table 1.

**Table 1:** Parameter descriptions, baseline values, and references

Description	Baseline value	Refs.
Average time a person remains in the non-infectious latency state ($\alpha$)	$lognormal(7,3)^{b}$	[25]
Proportion of asymtomatic people (*f*)	0.40	[26]
Average recovery time ($\gamma _1$)	15 days	[27]
Proportion of hospitalized people ($\sigma _1$)	0.23	[28, 29]
Median number of days from symptom onset to hospitalization ($\gamma _2$)	4 (1, 9) days	[30]
Median number of days of hospitalization ($\gamma _3$)	6 (3, 10) days	[30]
Percent that die among those hospitalized ($\sigma _2$)	11.8%	[31]
Shedding probability	0.38	[a]
Infection probability	0.38	[a]
Introduction probability	0.05	[a]
Assumptions for the scenarios		
Percentage of staff using PPE	90%	[a]
Percentage of residents using PPE	75%	[a]
PPE effect ($OR_{pi}$)	0.1467	[a] [32]
Detection probability	$80\%$	[a]
Percentage of staff tested	$90\%$	[a]
Percentage of resident tested	$33.3\%$	[a]
Frequency of testing	*Weekly*	[a]
Vaccine effect ($OR_{\upsilon }$)	0.0493	[a] [33]
Vaccine immunity duration	120 days	[a]
Distribution of the staff agent characteristics		
CN contacts per hour	$Multinom \sim (X_{0}=0.7,X_1=0.3)$	
RN contacts per hour	$Multinom \sim (X_0 = 0.25, X_1 =0.75)$	
LPN contacts per hour	$Multinom \sim (X_0=0.15,X_2=0.2,X_3=0.25, X_4 =0.2, X_5 =0.2)$	
Work schedule	$Multinom \sim (X_{morning}=0.4, X_{afternoon}=0.4, X_{night}=0.2)$	
Staff type	$Multinom \sim (X_{CN}=0.6, X_{RN}=0.15, X_{LPN}=0.15)$	

^a^Explored via sensitivity analysis

^b^Fitted to a distribution from data and truncated to a range of plausible values

### Disease dynamics

When an infectious individual contacts a susceptible, the transmission of the virus follows a Bernoulli distribution $P(y_i = 1 | x_j = 1) = Bernoulli(p_t)$. $y_i = 1$ represent the agent *i* getting infected, $x_j = 1$ represent the agent *j* being infectious and the parameter $p_t$ represent the probability of a transmission event happening. The parameter $p_t$ is estimated per agent, per time step of the simulation based on agent attributes as shown in the Eq. 1. We assume that there is a reduction in the probability of transmission from asymptomatic individuals, which was explored via sensitivity analysis. Due to default preventive testing and isolation measures, only infectious (both symptomatic and asymptomatic) agents that have not been detected and isolated may contribute to new infections. A newly-infected individual enters a latency period sampled from a lognormal distribution with a mean of 7 days [25]. After that time, the agent will become infectious, and 40% of people remain asymptomatic [26] until recovery. For those who develop symptoms, 23% [28, 29] require hospitalization. The average number of days from the onset of symptoms to hospitalization is 4 days and a person stays in the hospital for an average of 6 days [30]. Mortality rate was set at 11.8% [31] for hospitalized agents. The average recovery time for asymptomatic agents or those who never required hospitalization is 15 days [27], during which they remain infectious. Only residents are followed up after infection. Staff agents are assumed to leave during the infectious period and do not contribute further to disease spread. We assumed that recovery from a primary infection provided adequate immunity for the remainder of the simulation.

### Staff and resident interactions

Agents in the model include residents and staff only, consistent with the full visitor restrictions. Three residents are assigned to a single room. Five rooms are designated for quarantine/isolation of infected patients or for residents who require outside specialty care, such as dialysis. Residents only interact with two other residents in the same room and with staff, who can be one of three types: Certified Nursing Assistant (CNA), Registered Nurse (RN), and Licensed Practical Nurse (LPN). Isolated residents still interact with staff, but it is assumed that due to the behavioral changes, the transmission probability is reduced. Since meals are taken in rooms and use of communal space is restricted, residents do not currently interact with residents outside assigned rooms.

At the start of the simulation, the 170 staff members are assigned a staff type based on the distribution described by Table 1. Each type of staff has different contact patterns with residents throughout the day. These contact rates are operationalized as contact probabilities defined from a multinomial distribution where each hour a CNA has a 0.7 chance to have 0 contacts and 0.3 chance to have 1 contact with a resident, a LPN has a 0.15 chance of having 0 contacts, 0.2 of two contacts, 0.25 chance of having 3 contacts, and so on (Table 1). Contact probability parameters were estimated from staff hour-per-resident-day (HRD) data from the CMS Nursing Home Compare data set. All residents have equal probability of interacting with any staff member, staff agents will prioritize the residents with less number of contacts. We assumed no difference in probability of viral introduction by staff type. Staff are assigned to one of three different work schedules and will spend their time at the community otherwise; 40% work in the morning (7 am–3 pm), 40% in the afternoon (3–11 pm), and 20% work overnight. They spend on average 8 hours inside the nursing home and the rest of the time in the community. Both scheduled time and type of staff are sampled from a multinomial distribution to reflect the distribution in our reference nursing home (Table 1).

### COVID-19 transmission in the community

Though there is large variability on the impact of COVID-19 in these facilities, tied to historic variability in testing capacity and PPE availability and adherence, the most immediate risk of a COVID-19 outbreak in a nursing home is the level of community transmission of SARS-CoV-2. Since we assumed that visitors are disallowed completely, residents’ risk for primary exposure is contact through staff who acquired an infection from the wider community. A critical factor that our model aimed to study was to assess the impact of the probability of viral introduction from the community on the predicted size of internal outbreaks. Each scenario we investigated was simulated across three different probabilities of a staff member contacting with an infected individual at the community: low (1% per day), medium (5% per day), and high (10% per day). These are expressed as “introduction probability”, which is set to 0.05 for the baseline scenario 1.

### Interventions

We parameterized interventions with variable impacts on the transmission of SARS-CoV-2: PPE use and misuse, regular diagnostic testing, and vaccinations. We considered scenarios where staff were tested every 7 days (baseline), 5 days, and 3 days (universal testing). Testing of residents in all scenarios assume that one resident per room is tested weekly, systematically cycling through the each resident every 3 weeks. Reduction in transmission probability from PPE use and vaccination were applied by modifying the shedding and infection probability parameters (Additional file 1: Table S1). Vaccine efficacy was translated into odds ratios of infection given exposure from the Pfizer and Moderna phase 3 clinical trial results. For brand- and age-agnostic scenarios, including the baseline scenario, the crude overall odds ratio was set to 0.0493. In scenarios where vaccine brand and recipient age were taken into account, the efficacy of the Moderna vaccine after the second dose was 95.6% (OR 0.0441) for individuals under 65 years old and 86.4% (OR 0.1357) for 65 and older [33]. The efficacy of the Pfizer vaccine for individuals under 65 was roughly equivalent to Moderna (OR 0.434), but was 94.7% (OR 0.0619) for individuals 65 years and older [34]. For ease of implementation, residents were considered 65 and older, and staff were considered under 65. The vaccine odds ratio has a direct impact on transmission probabilities and reflects the upper bounds for vaccine efficacy according to Eq. 1. Let $p_t$ be the probability of a transmission event:

1 $$\begin{aligned} p_t = \dfrac{e^{\ln (OR_{\omega }X_{\omega })+\ln (OR_{\pi }X_{\pi })+\ln (OR_{\nu }X_{\nu })}}{1+e^{\ln (OR_{\omega }X_{\omega })+\ln (OR_{\pi }X_{\pi })+\ln (OR_{\nu }X_{\nu })}} \end{aligned}$$

where the odds ratio $\omega$ ($OR_{\omega }$) represents the global baseline transmission probability of all agents, the odds ratio $\pi$ ($OR_{\pi }$) represents the transmission reduction from the presence or absence of PPE, and the odds ratio $\nu$ ($OR_{\nu }$) corresponds to the effect of vaccine status on transmission. Probability $p_t$ is computed for all agents at each time step in order to reflect different probabilities of transmission based on the interventions each individual received. For scenarios where a vaccine was implemented, we specified the proportion of residents and staff that received a vaccine and a fixed time interval of 21 days between the first and second dose, with a 60% efficacy after the first dose but before the second.

The baseline scenario assumed the CDC infection prevention and control recommendations for nursing homes, including visitor restrictions, daily symptom screening of residents and staff, use of face masks, and weekly testing of staff. We incorporated weekly cyclic testing of one of three residents per room, with alternating residents being tested each week. When a resident tested positive, they were isolated and the other residents from the same room were tested. Staff who tested positive were “isolated” (removed from the simulation, as if on paid leave) and replaced with new staff who tested negative. Parameters assumed for the baseline scenario are described on Table 1.

## Model implementation

The model was implemented in GAMA 1.8.1 [35] and analysis were conducted in R [36]. Code for reproducing this study is available at https://github.com/jpablo91/NH_COVID. We ran 4000 simulations for simulated period of 150 days using a controlled random seed. To incorporate stochasticity, for each simulation run, we sampled a set of parameters from a list of possible values representing low, moderate, and high estimates (Table 2). The decision on the different parameter sample space was either changing (increasing or decreasing) in a 20% parameter estimate or based on the estimate and confidence interval reported in the literature. The magnitude of the outbreak was measured from the results recorded in the model, including: Time to disease elimination from the facility, attack rate, cumulative number of infected, hospitalizations, and deaths.

The model was calibrated and validated with data on confirmed COVID-19 cases reported between May 24, 2020, and February 14, 2021, in California nursing homes with similar resident census (between 150 and 200 occupied beds) extracted from the Centers for Medicare & Medicaid Services (CMS) [37]. Given the lack of literature to parametrize the transmission probability in detail, we adjusted this parameter until the model results were similar to the cumulative number of infected, for both residents and staff, from the observed outbreaks. For model validation, we compared the observed cumulative median of infected to the median estimated from the time steps in our simulation results of the baseline scenario using correlation analysis and fitted a simple linear regression. We considered as a good fit to have high $R^2$ and Pearson’s *R*.

### Analysis of outcomes and sensitivity analysis

We fitted simple linear regression models to estimate differences in the simulation outcomes to evaluate how sensitive our model is to the parameter changes. Random forest (RF), to rank the most influential parameters, and classification and regression trees (CART) to provide a graphical understanding of how parameters interact to affect the outcome, similar to the process described by [38] for global sensitivity analysis (GSA) of complex models.

**Table 2:** Parameters explored for global sensitivity analysis

Parameter name	Definition	Sample space
Infection_p	Global transmission probability	(0.304, 0.38, 0.456)
Introduction_p	Probability of introduction from the community	(0.01, 0.05, 0.1)
AsymptTransmission	Asymptomatic transmission	(0.34, 0.42, 0.99) [39]
SR_OR	Reduction on transmission when comparing staff to residents	(0.5, 0.7, 1.0)
TestingFreq	Frequency of testing (days)	(3, 5, 7)
detection_p	Probability of correctly identifying a COVID-19 positive case	(0.64, 0.8, 0.96)
PPE_OR	Influence of PPE in reduction of transmission expressed as odds ratios	(0.0722, 0.1467, 0.3408) [32]
VaccineEff	Vaccine efficacy	(Equal, A, B)
vaccination_dist	Distribution of the vaccine amongst residents and staff	(0.0, 0.3, 0.5, 0.7)

Equal vaccine efficacy assume that the efficacy was the same for resident and staff agents, A refers to a vaccine efficacy according to the reported by [33], and B a vaccine efficacy reported by [34]

## Results

**Fig. 2:**
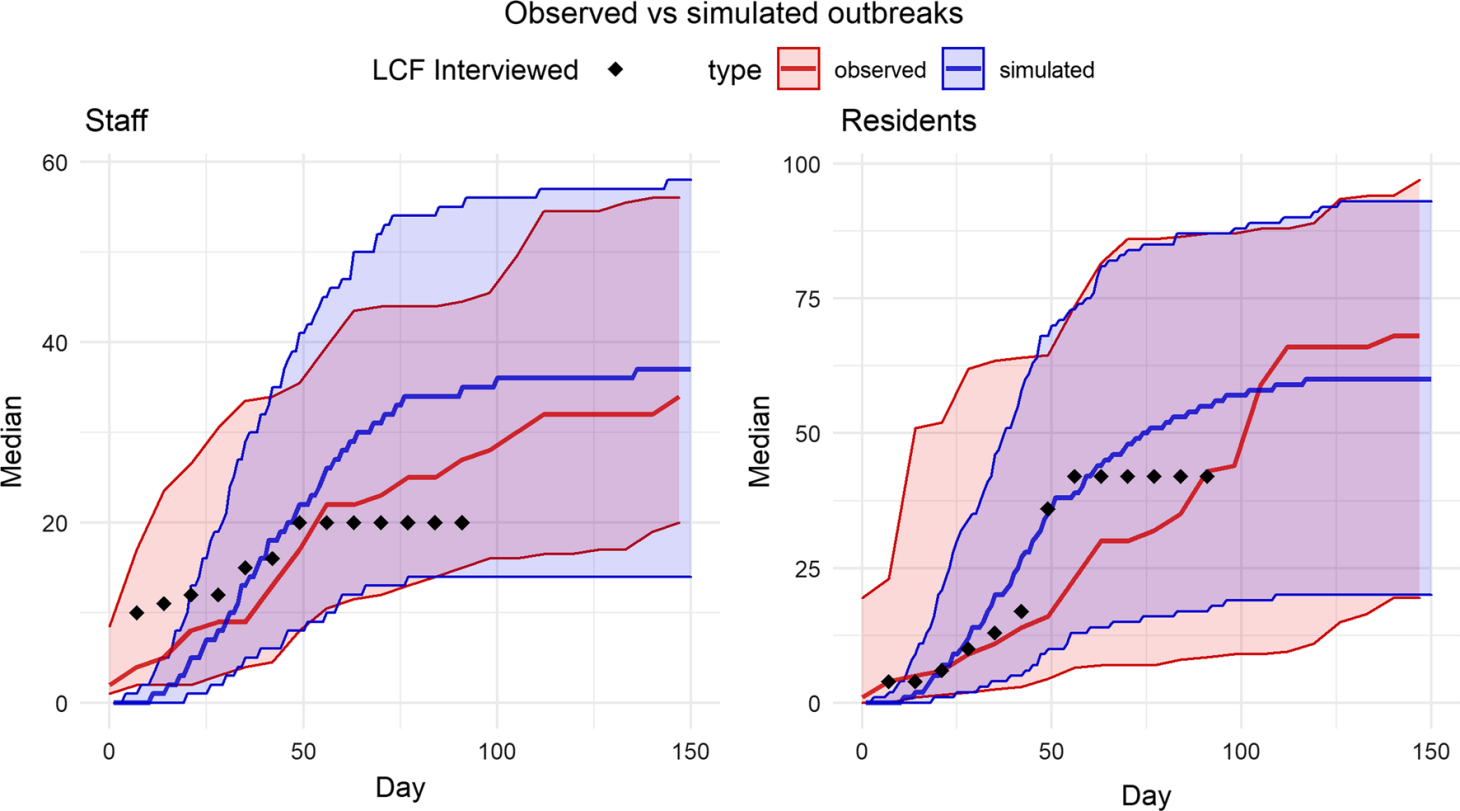
Model-predicted and observed number of cumulative incidence of confirmed cases for residents and staff. Dotted data represent the number of cases observed in the nursing home of study. Dark solid lines correspond to the median estimates for cases of staff and residents, and 25th and 75th percentiles are depicted in the shaded regions

The model was well calibrated to the cumulative number of cases among residents and staff in California nursing homes. When comparing simulated with the observed total infections, we estimated a Pearsons *R* of 0.93 and a $R^2$ of 0.87. Observed vs. expected median, 25th, and 75th percentiles for the cumulative number of expected and simulated infected staff and residents are shown in (Fig. 2). We included the number of cumulative infected from an outbreak that happened in the same facility that was interviewed to develop the model.

### Baseline scenario

In the baseline scenario, we assumed PPE mandates, weekly testing, and no vaccination. Baseline attack rate was 0.29 (95% CI 0.26, 0.31) and a median time to elimination from the facility of 106 days (95% CI 99.9, 112.88). With the implementation of the vaccine, under the assumption of equal vaccine efficacy for residents and staff, the attack rate decreases in average 0.16 (0.14, 0.17) and the time to elimination decreases in average 38.89 (35.4, 42.38) days in average (Table 3).

**Table 3:** Difference in simulation outcomes estimated by regression when compared to baseline

Parameter	Days to eradication	Attack rate	Total infected	Infected residents	Infected staff	Hospitalizations	Deaths
Baseline	106.46 (99.9, 112.88)	0.29 (0.26, 0.31)	98.5 (90, 106)	60 (54.5, 65)	38 (35, 41)	25.5 (23, 27.5)	3 (2.5, 3.5)
Transmission probability							
Low transmission (0.304)	$-$ 19.96($-$ 26.68,$-$ 13.24)*	$-$ 0.12($-$ 0.16,$-$ 0.09)*	$-$ 42.73($-$ 53.82,$-$ 31.65)*	$-$ 26.06($-$ 32.8,$-$ 19.31)*	$-$ 16.68($-$ 21.12,$-$ 12.23)*	$-$ 10.79($-$ 13.68,$-$ 7.9)*	$-$ 1.38($-$ 1.79,$-$ 0.97)*
High transmission (0.42)	9.81(2.95,16.68)*	0.11(0.07,0.14)*	36.4(25.08,47.71)*	21.83(14.94,28.71)*	14.57(10.03,19.11)*	9.27(6.32,12.22)*	1.13(0.72,1.55)*
Introduction probability							
Low introduction probability (0.01)	$-$ 26.82($-$ 33.71,$-$19.93)*	0($-$ 0.04,0.03)*	$-$ 0.28($-$ 12.44,11.89)*	$-$ 3.34($-$ 10.66,3.99)*	3.06($-$ 1.85,7.97)*	$-$ 1.56($-$ 4.69,1.57)*	$-$ 0.22($-$ 0.65,0.22)*
High introduction probability (0.1)	3.38($-$ 3.7,10.47)*	0.11(0.07,0.14)*	37.29(24.78,49.81)*	26.52(18.98,34.06)*	10.78(5.72,15.83)*	11.28(8.06,14.5)*	1.41(0.96,1.86)*
Detection probability							
Low detection probability (0.64)	10.16(1.68,18.64)*	0.03($-$ 0.01,0.07)*	10.81($-$ 3.15,24.78)*	7.9($-$ 0.54,16.34)*	2.92($-$ 2.75,8.58)*	3.54($-$ 0.09,7.17)*	0.3($-$ 0.23,0.82)*
High detection probability (0.9)	$-$ 23.49($-$ 30.17,$-$ 16.82)*	$-$ 0.19($-$ 0.22,$-$ 0.16)*	$-$ 65.23($-$ 76.23,$-$ 54.23)*	$-$ 40.11($-$ 46.76,$-$ 33.47)*	$-$ 25.12($-$ 29.58,$-$ 20.66)*	$-$ 16.49($-$ 19.35,$-$ 13.63)*	$-$ 1.86($-$ 2.27,$-$ 1.45)*
PPE effect							
High effect PPE (0.07)	$-$ 41.73($-$ 52.31,$-$ 31.14)*	$-$ 0.09($-$ 0.13,$-$ 0.04)*	$-$ 29.24($-$ 46.43,$-$ 12.05)*	$-$ 17.58($-$ 27.98,$-$ 7.17)*	$-$ 11.66($-$ 18.63,$-$ 4.7)*	$-$ 7.14($-$ 11.62,$-$ 2.66)*	$-$ 1.03($-$ 1.68,$-$ 0.37)*
Low effect PPE (0.34)	22.13(16.63,27.63)*	0.22(0.19,0.24)*	75.13(66.2,84.06)*	46.32(40.91,51.72)*	28.82(25.2,32.43)*	19.35(17.02,21.68)*	2.21(1.87,2.55)*
Asymptomatic transmission							
Low asymptomatic transmission (0.34)	21.44(12.12,30.76)*	0.08(0.04,0.13)*	29.14(13.41,44.86)*	18.27(8.75,27.79)*	10.86(4.52,17.21)*	7.7(3.63,11.77)*	0.89(0.31,1.47)*
High asymptomatic transmission (0.99)	26.85(18.19,35.52)*	0.23(0.19,0.27)*	78.69(64.06,93.32)*	49.4(40.54,58.26)*	29.29(23.39,35.19)*	21.18(17.4,24.96)*	2.24(1.7,2.77)*
Age specific difference in transmission							
High age specific difference (0.34)	$-$ 2.11($-$9.08,4.86)	0($-$ 0.04,0.03)	$-$ 0.33($-$12.47,11.8)	0.25($-$ 7.12,7.63)	$-$ 0.59($-$5.44,4.26)	0.14($-$ 3.01,3.29)	0($-$ 0.44,0.44)
Low age specific difference (0.99)	$-$ 4.64($-$ 11.56,2.28)	0($-$ 0.04,0.03)	$-$ 0.8($-$ 12.86,11.25)	$-$ 0.77($-$ 8.1,6.56)	$-$ 0.04($-$ 4.86,4.79)	$-$ 0.13($-$ 3.26,3)	0.01($-$ 0.43,0.44)
Testing frequency							
Testing frequency, 5-days^a^	$-$ 26.98($-$ 36.51,$-$ 17.44)*	$-$ 0.12($-$ 0.14,$-$ 0.1)*	$-$ 41.68($-$ 49.52,$-$ 33.84)*	$-$ 24.85($-$ 29.75,$-$ 19.95)*	$-$ 16.83($-$ 19.89,$-$ 13.77)*	$-$ 9.94($-$ 12.16,$-$ 7.72)*	$-$ 1.18($-$ 1.55,$-$ 0.82)*
Testing frequency, 3-days^a^	$-$ 48.53($-$ 58.31,$-$ 38.76)*	$-$ 0.17($-$ 0.19,$-$ 0.14)*	$-$ 57.33($-$ 65.37,$-$ 49.29)*	$-$ 34.57($-$ 39.59,$-$ 29.55)*	$-$ 22.76($-$ 25.9,$-$ 19.62)*	$-$ 14.59($-$ 16.86,$-$ 12.31)*	$-$ 1.71($-$ 2.08,$-$ 1.34)*
Vaccine distribution (when compared to no vaccination)							
Equal distribution	$-$ 38.89($-$ 42.38,$-$ 35.4)*	$-$ 0.16($-$ 0.17,$-$ 0.14)*	$-$ 53.66($-$ 58.48,$-$ 48.85)*	$-$ 33.29($-$ 36.21,$-$ 30.36)*	$-$ 20.37($-$ 22.34,$-$ 18.41)*	$-$ 13.76($-$ 15.01,$-$ 12.5)*	$-$ 1.65($-$ 1.82,$-$ 1.47)*
Resident priority	$-$ 34.08($-$ 37.67,$-$ 30.48)*	$-$ 0.15($-$ 0.17,$-$ 0.14)*	$-$ 52.95($-$ 57.92,$-$ 47.98)*	$-$ 36.16($-$ 39.18,$-$ 33.14)*	$-$ 16.79($-$ 18.82,$-$ 14.76)*	$-$ 15.05($-$ 16.34,$-$ 13.75)*	$-$ 1.82($-$ 2,$-$ 1.63)*
Staff priority	$-$ 42.09($-$ 45.62,$-$ 38.55)*	$-$ 0.16($-$ 0.18,$-$ 0.15)*	$-$ 56.14($-$ 61.02,$-$ 51.27)*	$-$ 32.45($-$ 35.41,$-$ 29.48)*	$-$ 23.7($-$ 25.69,$-$ 21.71)*	$-$ 13.66($-$ 14.93,$-$ 12.39)*	$-$ 1.7($-$ 1.88,$-$ 1.52)*
Vaccine effect (when compared to similar age-specific effect)							
Different age-specific vaccine efficacy [Pfizer] (0.06^b^,0.04^c^)	$-$ 1.12($-$ 4.41,2.17)	0($-$ 0.01,0.01)	$-$ 0.98($-$ 4.1,2.13)	$-$ 0.76($-$ 2.65,1.14)	$-$ 0.23($-$ 1.59,1.14)	$-$ 0.32($-$ 1.15,0.51)	$-$ 0.07($-$ 0.19,0.06)
Different age-specific vaccine efficacy [Moderna] (0.13^b^, 0.04^c^)	3.7(0.36,7.03)*	0.01(0,0.02)*	3.93(0.78,7.09)*	1.37($-$ 0.55,3.29)	2.56(1.18,3.95)*	0.58($-$ 0.26,1.43)	0.05($-$ 0.08,0.17)

*indicate significant difference

^a^no vaccination assumed

^b^Vaccine assumed for resident agents

^c^Vaccine assumed for staff agents

### Sensitivity analysis

**Fig. 3:**
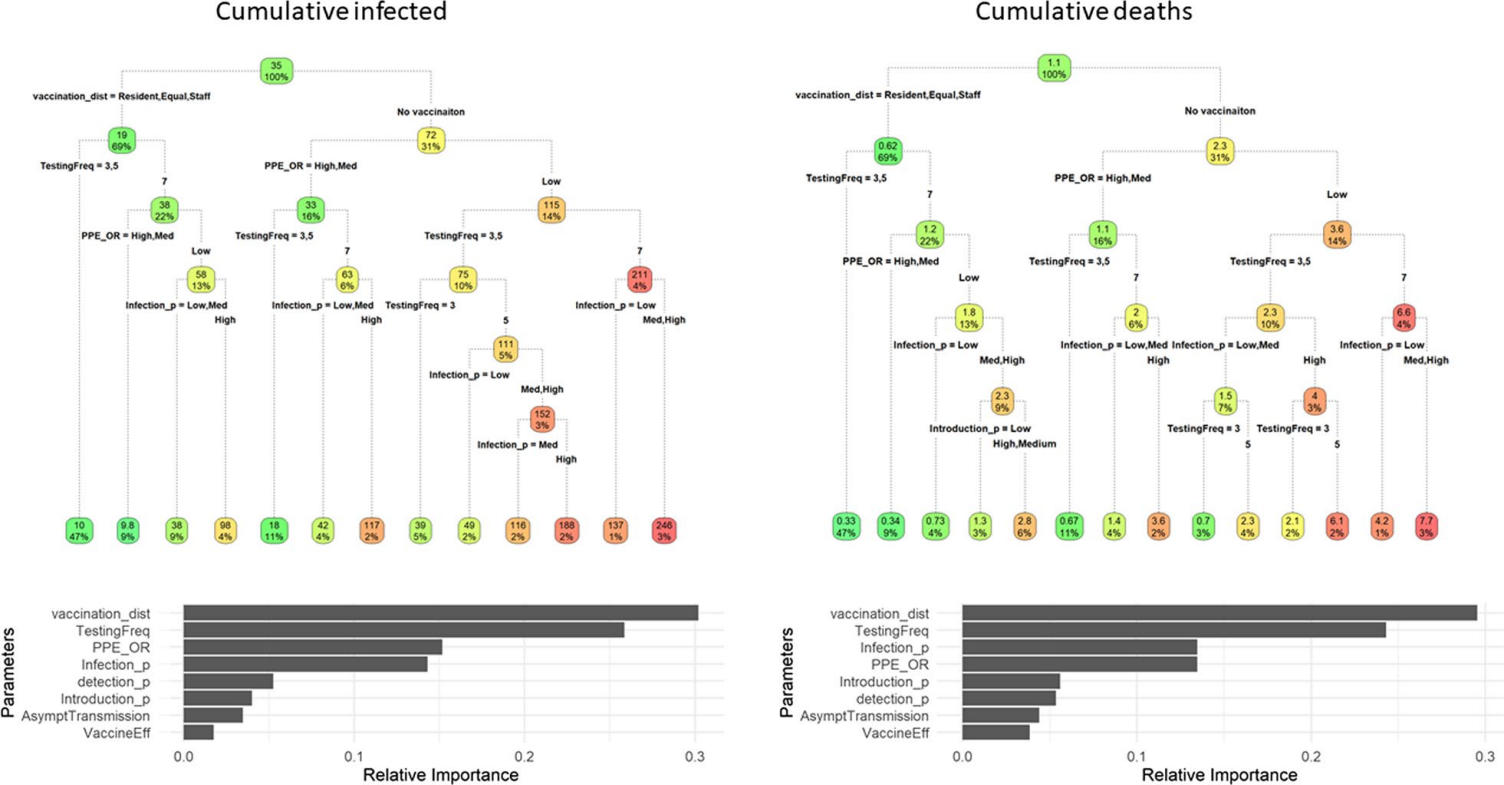
Global sensitivity analysis. Top, pruned regression trees that shows the interactions and different pathways of the simulation results spectrum. Bottom, variable relative importance estimated

RF and CART results show that the model predictions are sensitive to complex combinations of the parameter estimates. The influence the parameters and the interactions between them are presented in Fig. 3. For example, with the implementation of the vaccine and frequent testing, the expected number of infected was 10 (estimated from 47% of the simulations). But, when the vaccination was not implemented at all, the PPE was poorly implemented, testing was every 7 days, and the infection probability was high, the expected number of infected was 248 (estimated from 3% of the simulations) (Fig. 3). The classification and regression trees explained 83.98% and 61.6% of the cumulative infected and deaths variance, respectively. According to the RF results, the most influential parameters included vaccination (vaccine_dist), testing frequency (TestFreq), the effect of PPE (PPE_OR), and the infection probability (Infection_p). This was the same from both the cumulative number of infected and deaths, with the exception that for deaths, the infection probability was marginally more important than the PPE use.

### Testing and vaccine interventions

Vaccine implementation reduced the time to disease eradication in average 38.98 days (35.4, 42.38 95% CI), the attack rate in 0.16 (0.14, 0.17), hospitalizations in 13.76 (12.5, 15.01 95% CI) and deaths in 1.65 (1.47, 1.82).

The implementation of frequent testing, gradually reduced the expected cumulative incidence. Implementation of 5-day testing frequency reduced the average days to elimination in 26.98 (17.44, 36.51) and the attack rate in 0.12 (0.1, 0.14). When the testing frequency was done every 3 days, the days to eradication and attack rate were reduced in average 48.53 (38.76, 58.31) days and 0.17 (0.14, 0.19) (Table 3).

Some other parameters we examine had no statistically significant effects in our model, such as different age specific transmission.

**Fig. 4:**
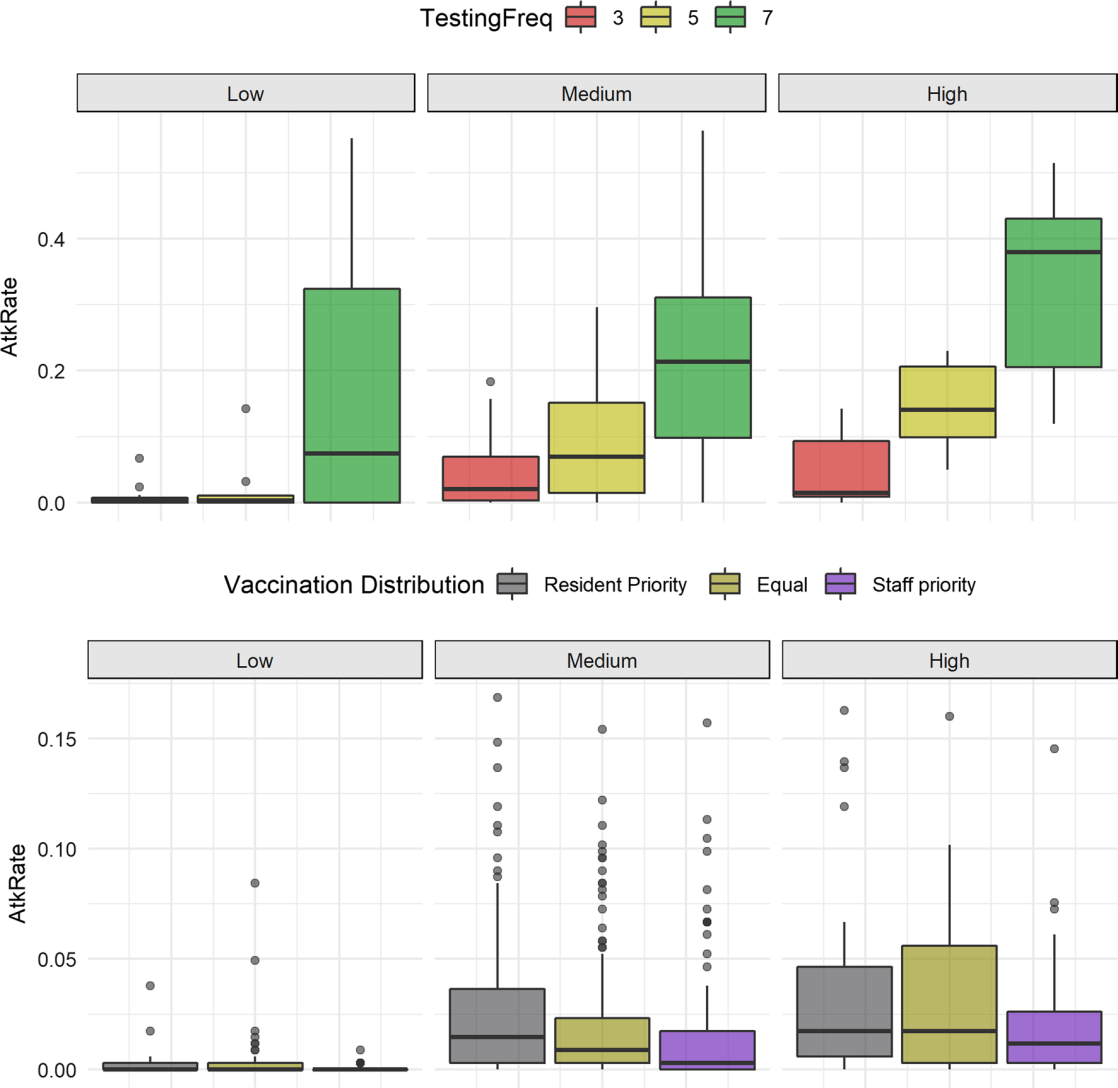
Attack rates for interventions under different assumptions of introduction probability (low, medium, high). Top presents scenarios for every 3, 5, and 7 days of testing. Bottom presents scenarios for the vaccine distribution for an equal distribution amongst residents and staff, staff priority and resident priority

To explore more in detail the role of testing at vaccination at different introduction probabilities, we fitted regression models adjusted for the different levels of community transmission. The difference in the simulation outcomes examined under these scenarios are presented in Table 4. The reduction in the outbreak magnitude with the interventions implemented was more evident when the probability of introduction was high, especially for the testing frequency (Fig. 4). For example, with a medium probability of introduction and 3-day testing, the attack rate was reduced in 0.17 (0.14, 0.19 95% CI); but for a high introduction probability, the 3-day testing reduced the attack rate in 0.29 (0.21, 0.37 95% CI). Prioritizing the vaccination for the staff members also had a greater effect on reducing the outbreak size. For example, with a high probability of introduction, when prioritizing the vaccination for the staff, there was a reduction in the days to eradication of 5.1 (0.4, 10.6 95% CI); however, when the vaccine was prioritized for the residents the hospitalizations were reduced in 3.27 (1.35, 5.18 95% CI).

**Table 4:** Results from scenario modeling using the median and 95% confidence intervals

Scenario	Days to eradication	Attack rate	Total Infected	Infected residents	Infected staff	Hospitalizations	Deaths
Introduction probability & testing frequency^a^							
High & 5-days	$-$ 11.22($-$ 34.84,12.4)*	$-$ 0.19($-$ 0.27,$-$ 0.11)*	$-$ 65.29($-$ 91.41,$-$ 39.16)*	$-$ 37.5(− 54.66,− 20.34)*	− 27.79(− 37.08,− 18.49)*	− 12.95(− 21.63,− 4.26)*	− 2.07(− 3.48,− 0.66)*
High & 3-days	$-$ 51.11($-$ 75.06,$-$ 27.16)*	$-$ 0.29($-$ 0.37,$-$ 0.21)*	$-$ 99.08($-$ 125.56,$-$ 72.59)*	− 59.23(− 76.63,− 41.83)*	− 39.85(− 49.27,− 30.43)*	− 24.07(− 32.87,− 15.26)*	− 3.08(− 4.5,− 1.65)*
Moderate & 5-days	$-$ 26.06($-$ 36.14,$-$ 15.98)*	$-$ 0.12($-$ 0.14,$-$ 0.1)*	$-$ 41.19($-$ 49.4,$-$ 32.97)*	− 24.86(− 30.02,− 19.71)*	− 16.32(− 19.51,− 13.14)*	− 10.16(− 12.49,− 7.82)*	− 1.19(− 1.57,− 0.8)*
Moderate & 3-days	$-$ 49.44($-$ 59.81,$-$ 39.07)*	$-$ 0.17($-$ 0.19,$-$ 0.14)*	$-$ 57.51($-$ 65.96,$-$ 49.06)*	− 35.06(− 40.36,− 29.75)*	− 22.45(− 25.73,− 19.18)*	− 14.9(− 17.31,− 12.5)*	− 1.75(− 2.15,− 1.36)*
Low & 5-days	$-$ 38.54($-$ 65.84,$-$ 11.23)*	$-$ 0.16($-$ 0.24,$-$ 0.07)*	$-$ 53.36($-$ 83.49,$-$ 23.23)*	− 29.8(− 46.98,− 12.62)*	− 23.56(− 36.66,− 10.46)*	− 13.76(− 21.93,− 5.58)*	− 1.31(− 2.48,− 0.15)*
Low & 3-days	$-$ 45.36($-$ 72.66,$-$ 18.05)*	$-$ 0.16($-$ 0.25,$-$ 0.07)*	$-$ 55.49($-$ 85.62,$-$ 25.36)*	− 30.53(− 47.71,− 13.36)*	− 24.96(− 38.06,− 11.86)*	− 13.89(− 22.07,− 5.71)*	− 1.51(− 2.68,− 0.35)*
Introduction probability & vaccine prioritization							
High & resident	2.01($-$ 3.61,7.62)	$-$ 0.01($-$ 0.03,0.01)	$-$ 3.26($-$ 10.11,3.59)	$-$ 6.79($-$ 11.16,$-$ 2.43)*	3.53(0.81,6.24)*	− 3.27(− 5.18,− 1.35)*	− 0.5(− 0.79,− 0.2)*
High & staff	$-$ 5.1($-$ 10.6, $-$ 0.4)*	$-$ 0.02($-$ 0.04,0)	$-$ 6.18($-$ 12.89,0.52)	$-$ 0.71($-$ 4.99,3.57)*	− 5.47(− 8.13,− 2.82)*	− 0.81(− 2.68,1.07)*	− 0.19(− 0.49,0.1)*
Moderate & resident	5.22(0.88,9.57)*	0($-$ 0.01,0.01)	$-$ 0.06($-$ 3.85,3.73)	$-$ 2.58($-$ 4.87,$-$ 0.3)*	2.53(0.89,4.16)*	$-$ 1.11(− 2.11,− 0.11)*	− 0.2(− 0.35,− 0.06)*
Moderate & staff	$-$ 2($-$ 6.89,2.9)	0($-$ 0.02,0.01)	$-$ 1.19($-$ 5.58,3.2)	2.02($-$ 0.62,4.66)	$-$ 3.21($-$ 5.12,− 1.31)*	0.74(− 0.42,1.9)	− 0.05(− 0.22,0.12)
Low & resident	1.96($-$ 3.52,7.45)	0.01(0,0.03)*	3.97($-$ 0.8,8.75)	0($-$ 2.45,2.45)	3.98(1.49,6.46)*	0($-$ 1.06,1.06)	0.16(0,0.32)
Low & staff	$-$ 6.36($-$ 11.74,$-$ 0.99)*	$-$ 0.01($-$ 0.02,0)*	$-$ 3.09($-$ 7.77,1.59)	$-$ 1.05($-$ 3.45,1.35)	− 2.04(− 4.48,0.4)	− 0.72(− 1.77,0.32)	0.01(− 0.15,0.16)

*Significant effect

^a^No vaccination assumed

## Discussion

The importance of careful use of non-pharmaceutical interventions was a critical lesson from the COVID-19 pandemic [40]. Mask policies, limited visitation, and especially frequent testing were critical to successful mitigation and prevention plans in the United States. Greater access to PPE and frequent testing surely played a part in reducing the case burden on LTCFs: case rates have dropped from a high of 33,625 nursing home cases/week to the current low of 1927 cases/week [37]. December 18, 2020 marked the start of the Pharmacy Partnership for Long-Term Care Program in which the CDC partnered with multiple pharmacies to host on-site vaccination clinics for LTCF residents and staff [8]. Despite good vaccination progress, nursing home residents remain at high risk. As regulations ease, and with the possibility of requiring yearly vaccinations to prevent future outbreaks, we must consider how surveillance, PPE usage, and vaccine timing and prioritization complement each other. Our study sought to describe the potential combined effects of recommended NPIs and vaccine deployment strategies on the size and duration of a COVID-19 outbreak in a model nursing home.

Prospectively, our model overestimated confirmed cases among staff, likely due the implementation of new interventions, like increased frequency of testing, put in place after SARS-CoV-2 was introduced in a nursing home. Our model underestimated cases among residents, which may be driven by the fact that some staff have more direct contacts with residents than others (Fig. 2).

Results from our model were most evident when we assumed a larger probability of viral introduction. In such cases, increased frequency of universal testing and isolation of positive cases lead to larger reductions in attack rate than any other scenario. Prioritizing the vaccination of staff over residents lead to a moderate decrease in attack rate, especially when viral introduction probability was high. Community transmission rate is the strongest predictor of case rates in nursing homes thus far [41] and staff are the most important vectors through which introduction from the community occurs [11, 12, 42]. Our results support using strategic prioritization of staff for universal testing, frequent testing of residents and vaccination as an important method for reducing the likelihood of an outbreak, especially in situations where community transmission is high.

There are several important challenges that these facilities will continue to face. LTCF administrators reported that staffing remains one of the primary barriers to maintaining high infection control standards [43]. Additionally, facilities that had a high degree of connectedness via shared staff showed higher case rates in general [44]. Expanded paid leave programs may be a solution to reduce the need for staff to seek additional employment to make ends meet, generally lowering their personal risk and the risk of introduction events.

Evidence indicates that staff may be more hesitant to get the vaccine than residents [10], and certainly have lower first-dose rates even if unrelated to hesitancy [8]. Vaccine mandates are one way to approach ensuring vaccine coverage goals are reached, but may create additional problems maintaining proper staff levels for delivering quality care. Additionally, nursing staff, including CNAs and LPNs, have high turnover rates in LTCFs. As a result, vaccination rates may fluctuate over time even within the same facility. Maintaining vaccine coverage goals will likely require an active program that includes acquiring confirmation from staff who receive vaccines from a different source (i.e. a local pharmacy or a different job). We have even less data about the risks presented by reopening nursing homes to visitors, prompting questions about vaccine and testing requirements for visitors. An extension to this model that adds a visitor agent could help answer these questions before observational data becomes available.

### Strengths and limitations

We calibrated our model using data from a real-world nursing home. The basal transmission model, in which no agents were vaccinated, generated plausible attack rates when compared to California nursing homes of a similar size. This, plus incorporating parameters from real-world data, provides external validity to the changes observed in our model. A particular strength of ABM is to show how complex outcomes can emerge from simple sets of rules; our model took advantage of this approach to show how interactions between staff and residents manifest the outbreak patterns observed in vivo. However, this model is primarily useful as an exploration of the impact of multiple interventions and introduction probabilities on an outbreak once introduction has occurred, and is therefore not meant to model the processes that lead to an introduction in the first place. Simulations were run for 150 days or until the facility was disease-free for up to 7 days; thus, it is also not able examine the impact of multiple introductions over longer periods of time or waning immunity from recovery or vaccination in its current form.

The behaviors of the agents in our model were designed based on the information provided by the nursing home interviewed during January 2021. With a rapid changing real world event such as the COVID-19 pandemic, some of these behaviors and the information used to parametrize this model might vary among different facilities and time periods. For example, the turnover of patients at the facility interviewed during the development of the model was very low, according to the nursing facility interviewed. This could have been different in other places. It is possible that the model presented here is not representing facilities where these recommendations were not followed. The model presented in this manuscript was specifically developed to explore the disease spread in the early stages of the vaccine distribution and implementation. Our ultimate goal was to develop a model that can be adapted to different settings, we hope we can keep updating and developing the model presented here to answer future research questions regarding the control and eradication of COVID-19 (and similar diseases) in LCTFs.

Not all data-derived parameters were made equally. The estimated effect of PPE use on transmission varied widely, thus making a reliable parameter difficult to define and the model sensitive to changes. Testing was also oversimplified in our model, as we assumed instantaneous results and all tests were equally sensitive. Additionally, we assumed that the effects of immunity, natural or from vaccination, was constant over the course of an outbreak and did not wane over time. We also assumed that staff agents had an equal chance of interacting with each resident agent, which is not reflective of intervention strategies that silo staff into daily routines focused on a specific subset of residents, such as dedicated staff for specific wards within the nursing home or for positive, isolated individuals.

## Supplementary Information

**Additional file 1: Table S1.** Agent attributes represented in the model. **Figure S1.** Epidemiological classes of the transmission model.

## Data Availability

The datasets generated and/or analysed during the current study are available in the github repository, https://github.com/jpablo91/NH_COVID. Data on case rates for California nursing homes were de-identified and publicly available from Centers for Medicare & Medicaid Services, available at: https://data.cms.gov/stories/s/COVID-19-Nursing-Home-Data/bkwz-xpvg
