# Supplementary material for: Testing and vaccination to reduce the impact of COVID-19 in nursing homes: an agent-based approach
Source: BMC Infect Dis. 2022 May 19;22:477. doi: 10.1186/s12879-022-07385-4 (PMC9117861; doi:10.1186/s12879-022-07385-4)
Supplement: Supplementary file 1 — Additional file 1: Table S1. Agent attributes represented in the model. Figure S1. Epidemiological classes of the transmission model. [file 12879_2022_7385_MOESM1_ESM.pdf]

Supplemental Material: Testing and Vaccination to Reduce the Impact of COVID-19 in Nursing Homes: An Agent-Based Approach

Model description.

The purpose of this model is to estimate the effect of vaccination and testing frequency of COVID-19 for disease control in a typical nursing home in California. We evaluate our model by its ability to reproduce observed patterns of disease spread on nursing homes in California with data from May 2020 to February 2021. The entities represented in our model include 170 residents, 174 staff members of the nursing facility. The agent attributes (state variables) are described in Table S1.
One time step represents one hour and simulations were run for a period of 150 days. The agents move in a continuous spatial surface bounded by the nursing home floor plans which includes the different rooms S1.
The simulation is initialized creating the agents with the attributes described by Table 1 from main manuscript. We assume that all resident rooms are full, so each room will create 3 residents. Attributes such as turn and type of staff, are assigned based on the multinomial distributions defined by the Table X of the main manuscript. All the resident and staff agents are initialized in the susceptible category for the disease status, and the schedule for every simulation step is described below:

- Infected agents execute their "Disease progression" sub-model, on which depending on the days since initial exposure, will transition trough the infection states represented S1 according to the rates specified by Table 1 from main manuscript.
- According to the testing schedule, all staff members and one resident per room will be tested for the disease. If an infected staff member is detected, the staff member will be sent back to the community for a period of 15 days and replaced with another staff. If an agent is detected positive, the other residents in the room will be also tested. All detected residents will be sent to one of the isolation rooms until tested negative.
- Agents will move randomly inside the current room, based on proximity and if the agent is infected, the transmission event will follow a binomial distribution $P(y = 1|x = 1) = p_t$, where $y_i = 1$ represents the event that agent i is infected, $x_j = 1$ represents that agent j is infectious, and p_t represent the probability of transmission estimated by the equation 1.
- If resident agents transition to hospitalization status, will be moved out of the facility to hospitalization until recovered or death, based on the rates described on Table 1 from main manuscript.
- According to their staff type, staff agents will randomly select a number of residents to contact. If the staff or agent is infected, the transmission event will follow a binomial distribution described by the transmission equation described before.
- Depending on the current simulation hour, the staff change will end their shift and go back to the community, next staff shift will start again. For each staff that returns from the community, the probability of introducing the disease is described by a Bernoulli distribution with $p =$ probability of introduction.

Stochasticity in our model plays an important role. Global parameters such as the transmission, introduction, vaccine efficacy and distribution, are randomly sampled from a parameter sample space at the initialization of the simulation. Behaviours of the agents such as movement are completely random, restricted by the nursing home floor plans. Other stochastic behaviours include the order of staff-resident

contacts, number of introductions to the nursing facility, testing, and transmission of the disease.
Interactions between the agents are defined based on spatial proximity and staff-to-resident contacts, which will influence the diseases spread. An example of a adaptive behaviour in our model is the staff-to-resident interactions, we assumed that all residents will be treated equally and the choice of which resident to contact in any given hour of the staff will be based on the number of previous interactions each resident agent has had during that turn, prioritizing the residents with least amount of contacts. Another adaptive behaviour in our model is the isolation of the infected agents, once detected the isolation of infectious agents will technically limits the disease spread.
For every simulation run, individual attributes such as the staff type, staff turn and adherence to PPE use

Table S1: Agent attributes represented in the model.

Name	Type	Dynamic	Range	Agent
Shift	Categorical	Yes	Morning, afternoon, night	Staff
Staff type	Categorical	No	CN, RN, LPN,	Staff
Infection probability	Continuous	Yes	0-1	Resident and staff
Disease status	Categorical	Yes	Susceptible, Infected, Infectious, Recovered, Hospitalized	Resident and staff
Days post exposure	integer	yes	0-inf	Residents and staff
At facilities	Boolean	Yes	true/false	Residents and staff
Vaccinated	Boolean	Yes	true/false	Residents and Staff
Days since vaccination	Integer	Yes	0-120	Residents and Staff
Number of contacts	Integer	Yes	0-Inf	Resident and Staff
Current room	Integer	Yes	Room 1- Room 60	Resident and Staff

Dynamic Transmission Model

Resident and staff agents in our proposed model are represented in epidemiological classes of susceptible but not yet exposed to the disease (S), non-infectious exposed individuals incubating the disease whose infection is currently non-detectable by testing (E), infectious individuals with detectable disease who do not yet exhibit clinical symptoms of illness (I_a), infectious individuals exhibiting symptoms of illness (I_s), individuals that have recovered and can no longer infect others (R), symptomatic individuals requiring hospitalization (H), and individuals that succumbed to the disease (D) (Figure 1). Transition parameters are described in Table 1 of main manuscript.

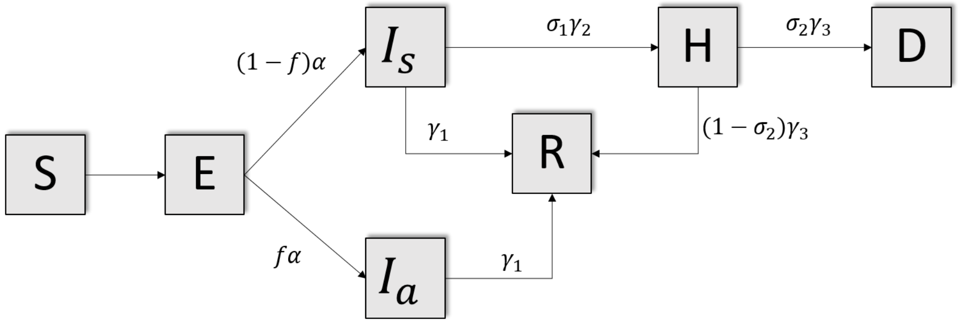

Figure S1: Epidemiological classes of the transmission model.
